# Complex PTSD symptoms predict positive symptoms of psychosis in the flow of daily life

**DOI:** 10.1017/S0033291724001934

**Published:** 2024-09

**Authors:** Peter Panayi, Emmanuelle Peters, Richard Bentall, Amy Hardy, Katherine Berry, William Sellwood, Robert Dudley, Eleanor Longden, Raphael Underwood, Craig Steel, Hassan Jafari, Richard Emsley, Liam Mason, Rebecca Elliott, Filippo Varese

**Affiliations:** 1 Division of Psychology and Mental Health, Manchester Academic Health Sciences Centre, University of Manchester, Manchester, UK; 2 Complex Trauma and Resilience Research Unit, Greater Manchester Mental Health NHS Foundation Trust, Manchester, UK; 3 Department of Psychology, King's College London, London, UK; 4 South London and Maudsley NHS Foundation Trust, London, UK; 5 Department of Psychology, University of Sheffield, Sheffield, UK; 6 Division of Health Research, University of Lancaster, Faculty of Health & Medicine, Lancaster, UK; 7 Department of Psychology, University of York, York, UK; 8 Psychosis Research Unit, Greater Manchester Mental Health NHS Foundation Trust, Manchester, UK; 9 Oxford Centre for Psychological Health, Oxford Health NHS Foundation Trust, Oxford, UK; 10 Oxford Institute of Clinical Psychology Training and Research, University of Oxford, Oxford, UK; 11 Department of Biostatistics and Health Informatics, Institute of Psychiatry, Psychology and Neuroscience, King's College London, London, UK; 12 Division of Psychology & Language Sciences, University College London, London, UK; 13 Division of Neuroscience and Experimental Psychology, University of Manchester, Manchester, UK

**Keywords:** disturbances of self-organization, ecological momentary assessment, experience sampling methodology, paranoia, trauma, visions, voices

## Abstract

**Background:**

Post-traumatic stress disorder (PTSD) has been shown to predict psychotic symptomology. However, few studies have examined the relative contribution of PTSD compared to broader post-traumatic sequelae in maintaining psychosis. Complex PTSD (cPTSD), operationalized using ICD-11 criteria, includes core PTSD (intrusions, avoidance, hyperarousal) as well as additional “disturbances of self-organisation” (DSO; emotional dysregulation, interpersonal difficulties, negative self-concept) symptoms, more likely to be associated with complex trauma histories. It was hypothesized that DSOs would be associated with positive psychotic symptoms (paranoia, voices, and visions) in daily life, over and above core PTSD symptoms.

**Methods:**

This study (*N* = 153) employed a baseline subsample of the Study of Trauma And Recovery (STAR), a clinical sample of participants with comorbid post-traumatic stress and psychosis symptoms. Core PTSD, DSO and psychosis symptoms were assessed up to 10 times per day at quasi-random intervals over six consecutive days using Experience Sampling Methodology.

**Results:**

DSOs within the preceding 90 min predicted paranoia, voices, and visions at subsequent moments. These relationships persisted when controlling for core PTSD symptoms within this timeframe, which were themselves significant. The associations between DSOs and paranoia but not voices or visions, were significantly stronger than those between psychosis and core PTSD symptoms.

**Conclusions:**

Consistent with an affective pathway to psychosis, the findings suggest that DSOs may be more important than core PTSD symptoms in maintaining psychotic experiences in daily life among people with comorbid psychosis and cPTSD, and indicate the potential importance of addressing broad post-traumatic sequelae in trauma-focused psychosis interventions.

## Introduction

Traumatic life experiences increase psychosis risk (Bell, Foulds, Horwood, Mulder, & Boden, 2019; Varese et al., 2012), and symptoms of post-traumatic stress mediate this relationship (Alameda et al., 2020; Sideli et al., 2020; Williams, Bucci, Berry, & Varese, 2018). In addition to the high rates of childhood trauma, people with psychosis also have a high risk of re-victimization that compounds the risk of symptoms of post-traumatic stress (De Vries et al., 2019). Comorbid post-traumatic stress disorder (PTSD) is therefore unsurprisingly common among people with psychosis, worsening mental health outcomes (DeTore, Gottlieb, & Mueser, 2021). However, it is unclear which post-traumatic sequelae account for the association between trauma and psychosis. The diagnostic conceptualization of PTSD has been subject to much debate, particularly regarding the extent to which it should focus on ‘core’ symptoms (i.e. intrusions, avoidance, hyperarousal) or those commonly associated with the complex trauma histories typically experienced by people with psychosis, conceptualized as disturbances of self-organization (DSOs; emotional dysregulation, interpersonal difficulties, negative self-concept) (Karatzias et al., 2017; Trauelsen et al., 2015). The newly classified diagnosis of complex PTSD (cPTSD) includes the core symptoms of PTSD as well as DSOs (Maercker et al., 2013).

Whilst the conceptualization of cPTSD in the latest International Classification of Disease (ICD-11; World Health Organisation, 2019) is relatively recent, existing findings on related concepts shed light on the role of DSOs in psychosis. Avoidant attachment, emotion dysregulation and negative self-beliefs have all been shown to predict positive symptoms, demonstrating a potential association between cPTSD and psychosis (Bloomfield et al., 2021; Hardy et al., 2016; Hardy, O'Driscoll, Steel, Van Der Gaag, & Van Den Berg, 2021; Sitko, Bentall, Shevlin, O'Sullivan, & Sellwood, 2014). Epidemiological studies indicate that a sizeable proportion of trauma survivors experience comorbid psychosis and cPTSD symptoms (Frost, Louison Vang, Karatzias, Hyland, & Shevlin, 2019). Preliminary studies suggest cPTSD may be more common than PTSD among people with psychosis, and potentially contribute to maintaining positive and affective symptoms (Panayi et al., 2022). Considering the impact of cPTSD may therefore prove essential to a comprehensive understanding of post-traumatic sequelae in people with psychosis.

One limitation of existing research on core PTSD and DSO difficulties in people with psychosis is the dominant use of cross-sectional designs (Bloomfield et al., 2021), since retrospective designs cannot establish temporal relationships and are subject to recall bias (Blum et al., 2015; Decker, Rosen, Cooney, Schnurr, & Black, 2021). In turn, existing longitudinal studies are typically aimed at detecting developmental change utilizing widely spread assessment points (Ram & Gerstorf, 2009), which may fail to capture the potential confounding effects of contextual factors (McNeish, Stapleton, & Silverman, 2017) and/or dynamic fluctuations in observed variables (Wang, Hamaker, & Bergeman, 2012). An examination of temporal dynamics using intensive longitudinal methods can address these limitations and shed light on the potential interrelatedness of cPTSD and psychosis in daily life.

Experience sampling methodology (ESM), a structured diary technique wherein participants are prompted multiple times per day to complete ambulatory assessments, can assess these temporal dynamics (Trull & Ebner-Priemer, 2009). ESM has increasingly been applied to study the flow and impact of post-traumatic symptoms in the daily lives of people with PTSD (Chun, 2016; Vachon, Viechtbauer, Rintala, & Myin-Germeys, 2019), as well as psychosis (Bell et al., 2024). Brand et al. (2020) investigated the daily impact of PTSD in people with psychosis, and did not find a temporal relationship between core PTSD symptoms and auditory hallucinations. The current study extends prior research by including wider post-traumatic sequelae – namely, DSOs – as well as a wider array of positive psychotic symptoms (i.e. paranoia, voices, and visions) to clarify previous cross-sectional relationships between DSOs and psychosis (Panayi et al., 2022) and aid the identification of further treatment targets of trauma-focused psychological interventions.

To the best of our knowledge, this study is the first to examine the dynamic effects of cPTSD on psychosis symptoms in people meeting ICD-10 criteria for schizophrenia-spectrum disorders and DSM-5 diagnostic criteria for PTSD. We aimed to examine the temporal association between DSOs and positive psychosis symptoms, accounting for concurrent PTSD symptoms. Based on cross-sectional findings that DSO-like difficulties mediate the relationship between childhood trauma and psychosis (Bloomfield et al., 2021; Sideli et al., 2020; Williams et al., 2018), it was hypothesized that daily increases in DSOs would predict subsequent exacerbations in psychotic experiences, and that this relationship would persist when controlling for core PTSD symptoms.

## Method

### Study design

This study used an ESM design (Myin-Germeys et al., 2018) involving the repeated assessment of positive psychosis symptoms (auditory and visual hallucinations; paranoia), PTSD (intrusions, avoidance, hyperarousal) and DSOs (emotional dysregulation, interpersonal difficulties, negative self-concept), using a mobile app that prompted participants to rate their experiences up to 10 times per day over 6 consecutive days.

### Participants

Participants (*N* = 153) were a subsample of the Study of Trauma and Recovery (STAR trial) (Peters et al., 2022) who consented to additional ESM procedures prior to randomisation. Participants met ICD-10 criteria for schizophrenia-spectrum diagnoses (F20–29) ascertained from the ICD-10 checklist by the research team, following clinical notes review and consultation with the care team, as appropriate, and scored ≥2 (‘moderate’ intensity) on the distress item of at least one psychotic symptom rating scale (see section 2.3.1) to ensure presence of at least one distressing positive symptom. Notably, 31% of the sample were recruited from Early Intervention for Psychosis (EIP) services, where United Kingdom (UK) good practice guidelines stipulate against assigning potentially stigmatizing diagnoses such as schizophrenia to those experiencing a first episode of psychosis, and instead routinely apply ICD-10 F28 (other non-organic psychotic disorder) or F29 (unspecified psychotic disorder) categories. The majority (114[75%]) had at least one other diagnosis, in addition to meeting criteria for psychosis and PTSD, with the most common being depression (58[38%]).

Participants also endorsed at least one traumatic life event on the Trauma and Life Events checklist (Carr, Hardy, & Fornells-Ambrojo, 2018), and met PTSD criteria on the Clinician-Administered PTSD Scale for DSM-5 (Weathers et al., 2018). Participants were excluded if they were <18, their psychotic or PTSD symptoms were primarily organic in etiology, had a primary substance misuse diagnosis, required an interpreter to engage with the trial, or (within the previous 3 months) had major medication changes or received trauma-focussed therapies.

Descriptive demographic and clinical variables are presented in Table 1, split by ESM participants and non-participants. ESM participants had significantly higher rates of ICD-10 F28 diagnoses, and non-participants significantly higher rates of ICD-10 F20 diagnoses. However, the groups did not differ significantly in severity of core PTSD, DSOs, paranoia, voices, or visions (test statistics presented in online Supplementary Table S1).

**Table 1.** Demographic and clinical variables for participants who accepted (*n* = 153) and declined (*n* = 152) ESM

Variable		ESM %	No ESM %
Gender	Male	39.22	44.08
	Female	56.86	55.26
	Non-binary	2.61	−
	Prefer not to say	1.31	0.66
Ethnicity	White British	73.86	73.03
	Black (African or Caribbean)	9.80	7.89
	Mixed heritage	4.58	5.92
	South Asian (Indian or Pakistani)	2.61	1.97
	Other	9.15	10.53
Relationship status	Single	58.17	65.13
	Cohabiting/Married/Civil partnership	30.07	21.71
	Separated/Divorced/Widowed	11.11	12.50
	Prefer not to say	0.65	0.66
Education	Primary education	2.61	2.63
	Secondary education	30.72	43.42
	Vocational education/college	39.22	26.61
	Higher education	22.88	19.08
	Other	2.61	0.66
	Prefer not to say	1.96	3.95
Employment status	Working	17.65	7.24
	Studying	4.58	5.92
	Volunteering	4.58	3.95
	Caregiver	−	1.32
	Retired	1.96	−
	Not currently working	69.93	82.24
	Prefer not to say	1.31	1.32
Schizophrenia spectrum diagnosis (F20-29; ICD-10)	Schizophrenia (F20)	15.03	23.68
	Persistent delusional disorder (F22)	1.31	1.32
	Schizoaffective disorder (F25)	9.80	15.79
	Other nonorganic psychotic disorder (F28)	30.72	17.76
	Unspecified nonorganic psychosis (F29)	43.14	41.45
Hears voices^a^		81.70	78.29
Sees visions^a^		63.40	53.95
Antipsychotic prescription		81.05	75.66
ITQ Diagnosis	None	8.55	14.09
	PTSD	8.55	8.72
	cPTSD	82.89	77.18
Other diagnoses	Anxiety disorders (Generalized anxiety, OCD, Other anxiety disorders)	15.69	19.08
	Autism	7.84	1.97
	Bipolar	6.54	9.87
	Depression (with or without psychotic features)	37.91	26.97
	Personality disorders (including emotionally unstable and other personality disorders)	30.07	22.37
	Substance-related disorders	6.54	9.21
	Other (e.g. ADHD, eating disorders, severe stress, and adjustment disorder)	9.80	12.50
Multiple trauma exposure	Repeated events (at least 1 TALE item endorsed ‘more than once’)	100	100
	Multiple trauma types	100	100
Trauma timing	Child (endorsed any TALE item < 16)	92.81	95.39
	Adult (endorsed any TALE item 16 or over)	95.42	94.74
	Both (endorsed any TALE item < 16 AND 16 or over)	88.24	86.84
		*M*(s.d.)	*M*(s.d.)
Age		37.00(12.14)	40.73(12.50)
Number of trauma types^b^		11.52(3.20)	11.16(3.12)
ITQ-PTSD		17.93(4.16)	17.34(4.51)
ITQ-DSO		18.63(4.52)	18.13(4.80)
GPTS-Persecution		23.25(11.38)	23.39(12.19)
PSYRATS-Visions		30.82(5.93)	30.24(6.26)
PSYRATS-Voices		30.81(5.88)	31.98(4.54)

ESM, Experience Sampling Methodology; M, Mean; s.d., Standard Deviation; PTSD, Post-traumatic Stress Disorder; DSO, Disturbances of Self-Organisation; ICD-10, International Classification of Disease 10th Ed.; ITQ, International Trauma Questionnaire (Cloitre et al., 2018); OCD, Obsessive Compulsive Disorder; ADHD, Attention Deficit and Hyperactivity Disorder; GPTS, Green et al., Paranoid Thoughts Scale (Freeman et al., 2021); PSYRATS, Psychotic Symptom Rating Scales (Haddock et al., 1999); TALE, Trauma and Life Events checklist (Carr et al., 2018).

a Based on baseline PSYRATS data.

b Based on baseline TALE data.

### Measures

#### Baseline measures

Participants completed the following instruments as part of a larger STAR trial baseline assessment battery (Peters et al., 2022):

- *Trauma and Life Events Checklist* (TALE; (Carr et al., 2018)): 22-item self-report checklist assessing difficult life experiences. Each event is rated according to its occurrence, whether this occurred repeatedly, and its timing (i.e. whether participants were under age 16, 16 or over, or both in instances of repeated events). The number of traumas endorsed may be summed to indicate the number of different types of traumatic experiences. The TALE demonstrates good test-retest reliability and convergent validity with related trauma measures (33).
- *International Trauma Questionnaire* (ITQ; (Cloitre et al., 2018)): 18-item self-report scale assessing presence and severity of PTSD and DSO symptoms within the past month. Both subscales comprise 3 symptom clusters, themselves composed of 2 items each, and 3 items capturing the functional impact of symptoms. Items are scored on a 5-point Likert scale from 0–4 (‘Not at all’–‘Extremely’). The ITQ diagnostic algorithm identifies a probable diagnosis of PTSD when a participant scores ≥2 on at least one item in each PTSD cluster, plus ≥2 on at least one functional impairment item associated with these symptoms. The cPTSD threshold includes that of PTSD plus a score of ≥2 on at least one item in each DSO cluster and of ≥2 on at least one functional impairment item associated with these symptoms. PTSD and DSO items were totaled to derive continuous severity scores from 0–24 on each subscale, with higher scores indicating higher severity. These were used to examine correlations between ESM and baseline measures of PTSD and DSOs. Both subscales demonstrate high internal consistency (both *α*'s ≥0.79; (Cloitre et al., 2018)).
- *Revised-Green et al., Paranoid Thoughts Scale* (R-GPTS; (Freeman et al., 2021)): 18-item self-report scale assessing paranoid ideation in the past month. Composed of two subscales, ideas of reference (8 items) and persecution (10 items), items are scored on a 5-point Likert scale from 0–4 (‘Not at all’–‘Totally’). Higher scores on both the reference (range 0–32) and persecution (range 0–40) subscale suggest higher intensity. Items on the GPTS-persecution subscale were totaled to examine correlations between ESM and baseline paranoia measures. Both subscales demonstrate high internal consistency (all *α*'s >0.9; (Freeman et al., 2021)).
- *Psychotic Symptom Rating Scales* (PSYRATS; (Haddock, McCarron, Tarrier, & Faragher, 1999)): structured clinical interview measuring positive symptoms of psychosis over the past month across two subscales. One measures frequency, intensity, impact and phenomenology of auditory hallucinations (11 items) and the other measures preoccupation, conviction, and impact of distressing beliefs (6 items). Items are scored on a 5-point Likert scale from 0–4, with anchors varying to suit each item; higher scores on both the voices (range 0–44) and beliefs (range 0–24) subscale suggest higher intensity. The PSYRATS displays good inter-rater and test-retest reliability (Drake, Haddock, Tarrier, Bentall, & Lewis, 2007). Average scores on the frequency and duration items of the auditory hallucinations PSYRATS were used to examine associations with the ESM voices item. The STAR trial added an adapted PSYRATs version capturing the presence of hallucinations in other modalities. Items, anchors, and scoring are identical to the voices subscale, but adapted to refer to non-auditory hallucinations (Tsang, 2023). Average scores on frequency and duration of visual hallucinations were used to examine associations with the ESM visions item

#### ESM measures

The ESM assessment comprised 29 items assessing affective states, contextual information, psychosis, and cPTSD symptoms. Items were scored on a 7-point Likert scale from 1–7 (‘Not at all’–‘Very much so’). Items were based on previous ESM studies of similar populations (Chun, 2016; Kimhy et al., 2006), amended in collaboration with consultants with lived experience of trauma and psychosis.

**Figure 1.**
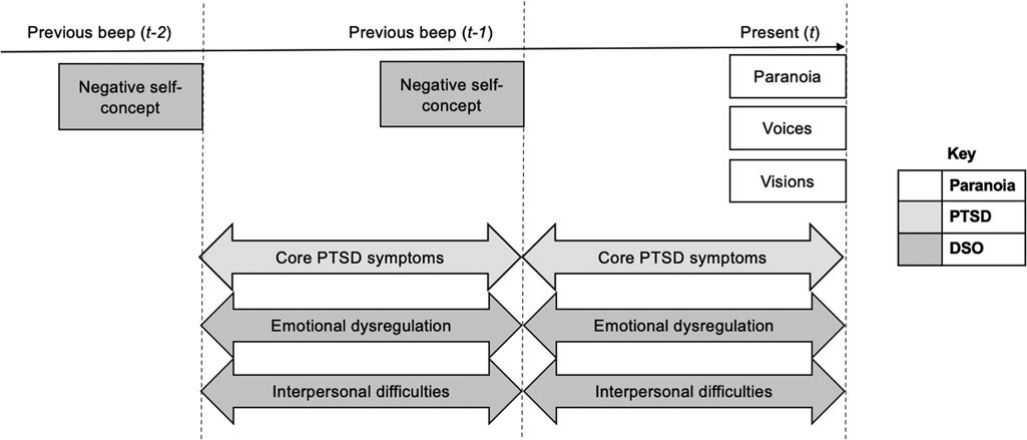
Temporal ordering of ESM items, adapted from (Palmier-Claus, Haddock, & Varese, 2019). Boxes indicate momentary items; arrows indicate interval items.

As illustrated in Fig. 1, some items (psychosis symptoms; negative self-concept [DSO]) were phrased to capture the current moment (*Right now…*, i.e. ‘momentary’ items), whereas others (core PTSD symptoms; emotional dysregulation [DSO]; interpersonal difficulties [DSO]) referred to the timeframe between moments (*Since the last beep…*, i.e. ‘interval’ items). We considered proximal models based on data from a single timepoint (time *t*) to nevertheless be longitudinal when interval items were included, since these analyses tested whether symptoms experienced since the previous moment (interval item scores) predicted symptoms at the current moment (momentary item scores). To further corroborate the temporal ordering of effects, we also assessed distal models using lagged predictor variables to test whether interval items at timepoint *t* − 1, and momentary items at *t* − 2, predicted momentary items at time *t*.

*Current psychotic symptoms:* Two items (*‘Right now I feel suspicious’;* ‘*Right now I believe that some people want to hurt me deliberately’*) were used to compute momentary paranoia scores. The mean of the two items was used, unless one was missing, in which case the remaining single item was used. Single items measuring voices (*‘Right now I hear a voice or voices that other people cannot hear’*) and visions (*‘Right now I see things that other people cannot see’*) were used to assess momentary hallucinatory experiences.

*Core PTSD and DSO symptoms:* An interval PTSD score was derived from the mean of five items across the three core PTSD symptom domains (intrusions; avoidance; hyperarousal). These items were anchored to the same traumatic experience(s) as the baseline STAR assessment (i.e. that which participants identified as affecting them the most recently). One item captured intrusive memories (‘*Unwanted memories about the experience popped into my mind’*), two measured avoidance (‘*I avoided thoughts, feelings and physical sensations that remind me of the experience’; ‘I avoided people, places or situations that reminded me of the experience’*) and two measured hyperarousal (*‘I felt super alert, watchful or on guard’; ‘I felt jumpy or easily startled’*). All five items were interval items (*‘Since the last beep…’*, capturing phenomena of interest that might have occurred in the time interval between the previous and current ESM time point) due to the possible insufficient occurrence of specific PTSD symptoms to enable sampling using momentary items (‘*Right now..*’).

Six items across the three DSO domains (emotional dysregulation; interpersonal difficulties; negative self-concept) were used to derive an interval DSO score. Two items measured emotional dysregulation (*‘I found it hard to control my emotions’; ‘I felt spaced out, numb or emotionally shut down’*) and another two measured interpersonal difficulties (*‘I felt distant or cut off from people’; ‘I found it easy to stay emotionally close to people’* [reverse-scored]). These items were phrased as interval items (‘*Since the last beep…’*), as the reflective nature of these constructs makes momentary evaluation difficult. The negative self-concept items, however, were momentary (*‘Right now, I feel ashamed’; ‘Right now, I believe I am a good person’* [reverse-scored]). An average of all six items would therefore have included momentary and interval items, which may not confer a valid estimate. To account for this, DSO scores were calculated using the mean of negative self-concept item scores from the preceding moment (*t* − 1*;* the so-called ‘lagged’ score) and of the emotional dysregulation and interpersonal difficulty item scores at time *t* (i.e. assessing the interval between the preceding and current moment), to capture DSO severity up to the current assessment point.

*ESM item validation:* Multilevel confirmatory factor analysis (mCFA) was conducted to verify the factor structure of multi-item ESM constructs whilst accounting for the hierarchical structure of the data (Forkmann et al., 2018). A three-factor solution representing paranoia, PTSD and DSOs demonstrated acceptable fit (CFI = 0.91; RMSEA = 0.05) (Forkmann et al., 2018) that was better (AIC = 203 555.14; BIC = 204 012.59) than a single-factor solution (AIC = 213 402.71; BIC = 213 825.15) at within- and between-participant levels.

Multilevel regressions were used to test whether cross-sectional scores on validated questionnaires at baseline predicted ESM measures of matching constructs. These confirmed that GPTS-persecution scores predicted daily paranoia scores (*b* = 0.043, *p* < 0.001, 95% CI 0.028–0.058)<TS: Please check for ‘95% CI’ here and elsewhere>. Additionally, frequency and duration items on the PSYRATS-voices and ‘other modalities’ subscales predicted daily voice and vision frequency among voice hearers and those who experienced visions, respectively (*b* = 1.174, *p* < 0.001, 95% CI 0.931–1.417; *b* = 1.005, *p* < 0.001, 95% CI 0.566–1.444). Similarly, ITQ-PTSD subscale scores predicted daily PTSD scores (*b* = 0.035, *p* = 0.004, 95% CI 0.011–0.059), and ITQ-DSO subscale scores predicted daily DSO scores (*b* = 0.025, *p* = 0.012, 95% CI 0.005–0.044).

Owing to the multilevel nature of ESM data, the restrictions placed by Cronbach's alpha on measures of internal consistency are too strict, and Macdonald's omega (*ω*) is preferred as a more robust estimate (Eisele, Kasanova, & Houben, 2022), yet the dual-item nature of paranoia in our ESM questionnaire prevented a valid calculation of *ω*. Cronbach's alpha was therefore used to conservatively estimate reliability in this sample, which was acceptable (*α* = 0.79). The internal reliability of ESM core PTSD and DSO measures was good (*ω* = 0.83 for both scales). Internal consistency estimates were not suited to the measurement of voices or visions, as they were measured by single items.

### Procedure

Participants were recruited from five National Health Service (NHS) Mental Health Trusts across the UK (NHS research ethics committee IRAS ID: 275697). After providing informed consent, participants referred to the trial completed an eligibility assessment administered by a trained research assistant (RA). Those eligible for the trial completed the standardized questionnaire and clinical interview measures used in this study (ITQ, PSYRATS, GPTS-R). The full STAR trial assessment battery and procedures are listed elsewhere (Peters et al., 2022). Following baseline assessment, those who consented to hear about additional studies within the trial were invited to take part in the ESM study, which took place prior to randomization to the trial arms.

The ESM study used a mobile app, m-Path (Mestdagh et al., 2023), to deliver ESM questionnaires up to 10 times a day for six days. Smartphones with preloaded SIM cards were provided for participants without access to either a smartphone or sufficient internet connection. To reduce burden, schedules were adapted to suit the waking hours of participants. Participants with an atypical schedule (e.g. an upcoming holiday or invasive medical procedure) were not onboarded to the study until after the event(s) had passed, to ensure study responses reflected a typical week for participants.

After providing informed consent, participants were supported by a RA to download m-Path onto their personal mobile phone or were provided with a phone for the duration of the study. Participants completed a practice ESM assessment with the RA to clarify understanding of the items and resolve any queries. Next, the RA scheduled up to 10 notifications – as many as the participants' waking hours would allow using 90-min increments – per day. Compliance with the study was monitored throughout, and a monitoring phone call made two days following the practice to ensure the app ran smoothly and items made sense. Participants who missed all notifications in a day were contacted to check for any issues with the app. Following completion of all six days, participants were debriefed, provided feedback on the study procedures, and reimbursed.

### Statistical analyses

Linear regression models are unsuitable for ESM data, due to its clustered nature (Carter & Emsley, 2019). Multilevel regressions extend linear models by allowing for variation within and between participants, thereby accounting for ESM clustering (Garson, 2013). This study employed two-level (observations nested within participants) as opposed to three-level models (observations nested within days nested within participants), as the latter has been shown to be suboptimal in the presence of autocorrelation (de Haan-Rietdijk, Kuppens, & Hamaker, 2016), which temporal networks suggest is typical of psychotic experiences (Contreras, Valiente, Heeren, & Bentall, 2020; Jongeneel et al., 2020). Multilevel models were estimated using the XTREG module in STATA 14 (Statacorp, 2015). Multilevel models report standardized regression coefficients (*b*); their relative sizes were compared using Wald tests of coefficient standard errors (Wald, 1943). As a dimensional experience, analyses involving paranoia included all participants (*N* = 153). Analyses involving hallucinations only included participants who heard voices (*n* = 125) or saw visions (*n* = 97), respectively.

Proximal analyses tested whether DSO symptoms since or at the previous moment (i.e. time *t* − 1 for momentary item scores [negative self-concept] and time *t* for interval item scores [emotional dysregulation and interpersonal difficulties]) predict current psychotic symptom scores at time *t*, and whether this association persisted when controlling for PTSD symptoms since the previous moment (i.e. time *t* interval item scores).

Distal analyses tested whether lagged DSO scores (i.e. time *t* − 2 for momentary items and time *t* − 1 for interval items) predict current psychotic experiences at time *t*, and whether this association persisted when controlling for PTSD symptoms at time *t* − 1 (interval items).

The sampling scheme was quasi-random (i.e. beeps sent at random points within each consecutive 90-min interval), meaning the interval between beeps varied. The maximum interval between beeps during which DSO and PTSD items were measured spanned 3 hours in proximal analyses (e.g. if time *t* was at the end of the 90 min block, and time *t* − 1 was at the beginning of the previous block), and 6 hours in distal analyses. The average intervals, however, were 90 and 180 min, respectively. Non-consecutive scores were used where immediately preceding observations were missing.

## Results

### Descriptive statistics

Of a total 8174 data points, 3.326 (40.69%) were missing. On average, participants were scheduled 54 beeps (maximum = 60) over 6 days and responded to 59.26% of these (*M* = 32; s.d. = 15). Participants who responded to any notifications were included in the analysis, since multilevel models are robust against unbalanced data, though 2.83% of questionnaires were responded to outside a predetermined 15-min window and were excluded from analyses (Delespaul, 1995). A multilevel model including consecutive beeps as a predictor of missingness suggested measurements were increasingly more likely to be missed as the assessment period progressed (OR = 1.01, *p* < 0.001, 95% CI 1.00–1.01).

An experience was considered present if a participant rated ≥2, since 1 was anchored to ‘not at all’. Any paranoia item was endorsed at 4295 (93.03%) timepoints; voices and visions at 3077 (66.65%) and 2580 (55.88%) timepoints, respectively. Any core PTSD symptom was endorsed at 4397 (95.24%) timepoints, and any DSO at 4600 (99.63%) timepoints. Within-participant means and standard deviations of ESM variables are listed in Table 2.

**Table 2.** Means (*M*) and standard deviations (s.d.) of within-participant means and within-participant standard deviations for all ESM variables

Variable		*M*(s.d.)	*MSD*(s.d.)
PTSD	Memory intrusions	4.14 (1.41)	1.49(0.64)
	Avoidance	4.30 (1.51)	1.10(0.54)
	Hyperarousal	4.11 (1.39)	1.010(0.57)
DSO	Emotional dysregulation	4.10 (1.32)	1.06(0.47)
	Interpersonal difficulties	4.61 (0.97)	1.03(0.43)
	Negative self-concept	3.78 (1.29)	0.88(0.42)
Psychosis	Paranoia	4.24 (1.44)	0.93(0.50)
	Voices	3.80 (2.11)	0.97(0.75)
	Visions	3.07 (1.96)	0.92(0.71)

*Note:* All variables were measured using a 7-point Likert scale (range 1–7). *M* (s.d.) refers to means of each item across participants, and s.d.s of those mean scores between participants. MSD (s.d.) refers to the mean s.d. of each participant's own mean, and the s.d. between participants.

### Proximal analyses

Multilevel linear regression models indicated that DSO symptoms since or at the previous moment significantly predicted paranoia, voices, and visions at the current moment. This effect of DSOs persisted for all three outcomes when including core PTSD symptoms since the previous moment, themselves also predicting all three outcomes. Wald tests suggested DSOs were a significantly better predictor of paranoia, but not voices or visions, than core PTSD symptoms. Regression and Wald test statistics are listed in Table 3.

**Table 3.** Regression statistics of multilevel models

Analysis	Outcome	Predictor(s)	*b*	95% CI	Wald test statistic
Proximal	Paranoia (*n* = 153)	Step 1			
		DSO	0.662***	0.618, 0.706	
		Step 2			5.55**
		DSO	0.457***	0.408, 0.505	
		PTSD	0.285***	0.251, 0.319	
	Voices (*n* = 125)	Step 1			
		DSO	0.639***	0.576, 0.701	
		Step 2			0.82
		DSO	0.382***	0.311, 0.453	
		PTSD	0.333***	0.146, 0.382	
	Visions (*n* = 97)	Step 1			
		DSO	0.482***	0.413, 0.551	
		Step 2			0.04
		DSO	0.293***	0.183, 0.343	
		PTSD	0.275***	0.221, 0.328	
Distal	Paranoia (*n* = 153)	Step 1			
		DSO	0.392***	0.334, 0.451	
		Step 2			2.52*
		DSO	0.283***	0.216, 0.350	
		PTSD	0.149***	0.103, 0.196	
	Voices (*n* = 125)	Step 1			
		DSO	0.294***	0.213, 0.375	
		Step 2			0.29
		DSO	0.182***	0.087, 0.277	
		PTSD	0.144***	0.079, 0.209	
	Visions (*n* = 97)	Step 1			
		DSO	0.235***	0.144, 0.325	
		Step 2			0.10
		DSO	0.142**	0.035, 0.248	
		PTSD	0.080**	0.045, 0.187	

*Significant at *p* < 0.05 level; **at *p* < 0.01 level; ***at *p* < 0.001 level.

### Distal analyses

Multilevel linear regression models assessing whether lagged DSO scores (described above) predicted positive psychosis symptoms at the current moment, and whether this association persisted when controlling for lagged PTSD symptoms, showed the same pattern of findings as the proximal analyses. Wald tests suggested that DSOs were a significantly stronger predictor of paranoia, but not voices or visions, than core PTSD symptoms. Regression and Wald test statistics are listed in Table 3.

## Discussion

We aimed to ascertain the relationship between cPTSD and psychosis symptoms in the flow of daily life in psychosis individuals who meet criteria for comorbid PTSD. Consistent with our hypotheses, the key findings indicate that both proximal and distal fluctuations in DSOs predicted momentary measures of paranoia, voices, and visions. These temporal associations persisted when controlling for fluctuations in core PTSD symptoms within the same timeframe. Furthermore, they were stronger than those between core PTSD and psychosis symptoms, which were themselves significant, particularly for paranoia.

To our knowledge, this is the first study demonstrating the impact of cPTSD in the daily life of people with psychosis. Our findings imply that cPTSD may play a key role in maintaining psychotic experiences in daily life. In particular, emotional and interpersonal difficulties, and negative self-concept, seem to have a greater influence on psychosis symptoms than traumatic memory intrusions, hypervigilance, and avoidance. These results align with cross-sectional research demonstrating the impact of complex trauma, PTSD, and prevalence of cPTSD in people with psychosis (Campodonico, Varese, & Berry, 2022; Panayi et al., 2022). They further extend those of previous ESM studies demonstrating the daily impact of childhood trauma and core PTSD in people with psychosis (Brand et al., 2020; Dokuz, Kani, Uysal, & Kuşcu, 2022) to include DSOs. In turn, our findings also align with prior ESM studies investigating the impact of constructs consistent with DSOs; for instance, momentary attachment insecurity (consistent with interpersonal difficulties) and emotional instability (consistent with emotional dysregulation) have been shown to increase subsequent paranoia (Nittel et al., 2018; Sitko, Varese, Sellwood, Hammond, & Bentall, 2016). The distal analyses carried out in this study confirmed that the short-term effect of DSOs on psychosis revealed by proximal analyses persists, albeit with smaller effect size, for a period of up to 6 hours. Significant relationships over this period suggest symptoms of cPTSD potentially earlier on in a day may affect subsequent psychotic experiences (especially for paranoia, where our confidence intervals are more robust).

The findings are aligned with an affective pathway to psychosis and multifactorial accounts of trauma in psychosis (Hardy, 2017; Morrison, Frame, & Larkin, 2003). Particularly, they highlight how negative self-beliefs, relationship difficulties, and emotion regulation may be important treatment targets alongside core symptoms of PTSD, since the effect of DSOs was comparatively larger than that of core PTSD symptoms for all positive psychosis symptoms (significantly so for paranoia). Novel statistical innovations may be used to extend the present findings by exploring the symptom overlap between cPTSD and psychosis, and by identifying directed paths between these clusters to support the development of trauma-focused psychosis interventions (e.g. using network analysis; Contreras et al., 2020).

There were a number of limitations in this study. Interval ESM items are standard in ESM questionnaire design (Eisele et al., 2022), but their use to make longitudinal assumptions could be questioned, since retrospective items could be influenced by current states and/or recall bias. However, DSOs by nature are psychologically reflective, such as emotional dysregulation (‘*Since the last beep, I found it difficult to control my emotions*’) and may occur infrequently enough that momentary items risk experiences being missed, such as interpersonal difficulties not arising due to a participant being alone (Eisele et al., 2022). Furthermore, the distal analyses findings mirrored those of the proximal analyses, providing strong evidence of a temporal relationship between DSOs and psychotic symptoms.

While a strength of this study was the inclusion of both visions and voices, the reliability of single items to measure them is questionable (Eisele et al., 2022). Hallucinations are multidimensional experiences, with disparate temporal dynamics for differing dimensions (Bless et al., 2020). Further, visual and auditory hallucinations can be difficult to distinguish from dissociative post-traumatic flashbacks (Wearne et al., 2022). Future studies should assess how different aspects of psychotic experiences (e.g. content, appraisal, impact/distress) may be affected by DSOs, and capture these experiences using multiple items to maximize precision.

ESM completion rates in this study may appear low at an average of 59%, indicating potential limitations with the sampling scheme. However, 6 days are considered a standard measurement window in ESM studies and participant-level variables more typically predict non-response in ESM than design characteristics (Rintala, Wampers, Myin-Germeys, & Viechtbauer, 2020; Vachon et al., 2019; van Berkel et al., 2020). Indeed, owing to the complexities of research engagement among people with psychosis, data attrition is more common than in control samples. The completion rates in our sample are typical of other ESM datasets in people with psychosis (Bell et al., 2024).

The majority of our sample (79%) met criteria for cPTSD on the ITQ (Cloitre et al., 2018). Our findings demonstrating a stronger relationship between DSOs and psychosis symptoms than those with core PTSD symptoms may have been a result of the preponderance of people with cPTSD relative to PTSD. Nevertheless, this imbalance is consistent with other trauma-exposed samples of people with psychosis (Panayi et al., 2022), and is typical of individuals presenting to mental health services, who overwhelmingly have complex trauma histories (Trauelsen et al., 2015). Additionally, 30% of our sample presented with personality disorder diagnoses alongside higher rates of ‘other non-organic psychosis’ than ESM non-participants. Since there were no significant differences in symptom severity between groups, our sample is likely representative of people with comorbid psychosis and PTSD symptoms. This includes those in EIP services, who show similar rates of clinically significant personality disorder traits (Archer, Shnyien, Mansfield, & Draycott, 2023), making our findings relevant to psychological interventions offered for this population.

Limitations notwithstanding, there are several implications of these findings. The potentially maintaining role of DSO symptoms supports current trauma-focused therapy practices involving stabilization to establish emotion regulation and build positive relationships, such as trauma-focused cognitive-behavioral and eye-movement desensitization and reprocessing therapies (Keen, Hunter, & Peters, 2017; Peters et al., 2022). Therapeutic approaches aimed at addressing intra- and interpersonal wellbeing, such as compassion-focused techniques (Millard, Wan, Smith, & Wittkowski, 2023), may also be a promising avenue for the ongoing development of trauma-focused psychosis interventions, particularly for those who may find trauma reprocessing intolerable (Lewis, Roberts, Gibson, & Bisson, 2020). Our findings also suggest that the assessment of DSOs may be highly relevant for subgroups of people with psychosis and complex trauma histories. This assessment should be sensitive to the potential difficulties associated with cPTSD, including establishing social support and emotional regulation to manage distress associated with trauma disclosure (UK Psychological Trauma Society, 2017). Lastly, the large proportion of participants in our sample endorsing visual hallucinations suggests the assessment of hallucinations in modalities other than auditory could be valuable.

To conclude, this study highlights the profound impact of cPTSD on people with psychosis. Specifically, DSO symptoms (i.e. emotional dysregulation, negative self-concept, and interpersonal difficulties) may maintain psychosis symptoms in the flow of daily life to an even greater extent than core PTSD symptoms. In turn, the exacerbation of distressing psychosis symptoms is in addition to the direct impact of these difficulties on individuals' quality of life and daily functioning. Future research is required to delineate relationships between specific symptoms of psychosis, PTSD and DSOs, as well as mechanisms by which this impact occurs. There are clear clinical implications to this research, namely confirming the need for incorporating therapeutic practices aimed at addressing DSOs in trauma-focused psychosis interventions.

## Supporting information

Panayi et al. supplementary material Panayi et al. supplementary material

## Supplementary material

The supplementary material for this article can be found at https://doi.org/10.1017/S0033291724001934.

## Collaborators

**Affiliated with South London and Maudsley NHS Foundation Trust**

Lucy Ring

Christopher Shoulder

Morwennna Rickard

Firat Denizcaglar

Kavita Bains

Freya Meyer

Leilia Jameel

Majella Byrne

Nadine Keen

Sarah Swan

Rachel Frost

Ze Freeman

Gary Ngai

Jess Bird

Constance Harvey

Edward Bickers

Kaisha Santosa

Samantha Mansell

Siamara Goddard

Molly Bird

Harriet Mellotte

Rhea Kohli

Katja Shultz

Antonia Obele

Elizabeth Leahy

Max Dormitontov

Faiza Abbaz

Ho Chak (Milton) Suen

**Affiliated with King's College London**

Margaret Heslin

Emma Tassie

Sarah Byford

Elizabeth Kuipers

Laura Potts

Inez Verdaasdonk

**Affiliated with Greater Manchester Mental Health NHS Foundation Trust**

Tony Morrison

Wendy Jones

Eleanor Longden

Aqsa Choudary

Marina Sandys

Kim Towey-Swift

Elizabeth Murphy

Vicky Brooks

Samantha Bowe

Alice Newton-Braithwaite

Elliot Brewer

Leah Orme

**Affiliated with University of Manchester**

Tony Morrison

**Affiliated with Cumbria, Northumberland, Tyne, and Wear NHS Foundation Trust**

Rebecca Miskin

Laura McCartney

Marsha Cochrane

Antonia Newman

Sarah White

Nina Cioroboiu

Louise Prentice

Jane Mitchell

Doug Turkington

Kevin Meares

Libby Oakes

**Affiliated with Oxford Health NHS Foundation Trust**

Amy Langman

Emily Smyth

Georgina Kirtland

Natalie Barnes

Kate Costello

Collins Larrys

Iulia Grabovschi

Charlotte Sagnay de la Bastida

**Affiliated with Sussex Partnership NHS Foundation Trust**

Kathryn Greenwood

Cat Sacadura

Sarah Mansfield

Ann Steele

Lauren Edwards

Dan Elton

Angie Culham

Nick Grey

Molly Heeger

Lucy Fisher

Krutika Sharma

Chris Scane

Lisa Wood

Joseph Sherborne

Lauren Mose

Guy Emery

Aparajita Pandey

**Affiliated with University of Sussex**

Kathryn Greenwood

David Fowler
