# Supplementary material for: Complex PTSD symptoms predict positive symptoms of psychosis in the flow of daily life
Source: Psychol Med. 2024 Oct 4;54(12):3489–500. doi: 10.1017/S0033291724001934 (PMC11496218; doi:10.1017/S0033291724001934)
Supplement: Panayi et al. supplementary material [file S0033291724001934sup001.docx]

**Supplementary Table 1.** Independent samples *t-*tests of differences in symptom severity between ESM takers and non-takers

| Variable | *t*(df) | *p* |
| --- | --- | --- |
| ITQ-PTSD | -1.184(299) | .238 |
| ITQ-DSO | -.938(299) | .349 |
| GPTS-Persecution | .131(280) | .896 |
| PSYRATS-Visions | -.534(116) | .594 |
| PSYRATS-Voices^a^ | 1.636(238) | .103 |

*^a^Equal variance not assumed*

*^Note; t =^* ^test statistic;^ *^df =^* ^degrees of freedom;^ *^PTSD = Post-traumatic Stress Disorder; DSO = Disturbances of Self-Organisation; ITQ = International Trauma Questionnaire^* ^(Cloitre et al., 2018)^*^; GPTS = Green et al Paranoid Thoughts Scale^* ^(Freeman et al., 2021)^ *^; PSYRATS = Psychotic Symptom Rating Scales^* ^(Haddock et al., 1999)^
